# Ectoparasites of brown rats (*Rattus norvegicus*) in Grenada, West Indies

**DOI:** 10.14202/vetworld.2019.1390-1394

**Published:** 2019-09

**Authors:** Katelyn Noelle Thille, Nia Francesca Rametta, Daniel Mark Fitzpatrick, Camille Coomansingh Springer, Keshaw Tiwari, Rhonda Denise Pinckney, Ravindra Nath Sharma

**Affiliations:** Department of Pathobiology, School of Veterinary Medicine, St. George’s University, Grenada, West Indies

**Keywords:** brown rats, ectoparasites, Grenada

## Abstract

**Background and Aim:**

Arthropod ectoparasites (mites, lice, ticks, and fleas) on common house rats or brown rats (*Rattus norvegicus*) are known to transmit zoonotic pathogens and diminish rat health. To the best of our knowledge, there is no published information regarding the prevalence of ectoparasites on *R. norvegicus* in Grenada. This study aimed to determine the prevalence and types of ectoparasites present on brown rats from Grenada.

**Materials and Methods:**

One hundred sixty-eight rats were collected live from the parishes of St. George and St. David, Grenada, from May to July 2017. Following euthanasia, external parasites were collected using fine combs, thumb forceps, and paper tape. Tape samples and free specimens were placed in containers with 70% ethanol. External parasites were evaluated using dissection microscopy.

**Results:**

Over 2000 ectoparasites were collected from 149 of the 168 trapped rats (88.7%). Ectoparasites identified included mesostigmatid mites (found on 84.6% of infested rats), fur mites in the families Atopomelidae and Listrophoridae (67.1%), *Polyplax* spp. lice (6.7%), fleas (3.4%), an unidentified larval tick (0.7%), and a mite in the family Myobiidae (0.7%). Infestation rates were higher in St. David Parish (86/89; 96.6%) than in St. George Parish (63/79; 79.7%) (p=0.001). When comparing sex and age, males had a marginal increase in infestation rate (83/89; 93.3%) compared to females (66/79; 83.5%) (p=0.054), and adults had an infestation rate of 90.7% (97/107) compared to juvenile rats who had a 66.7% (14/21) infestation rate.

**Conclusion:**

Brown rats in Grenada are heavily infested with ectoparasites, including known vectors of pathogens. Future studies will examine the prevalence of zoonotic pathogens in these arthropods.

## Introduction

*Rattus norvegicus*, the common house rat, can be found across the world densely populated in temperate climates. Rodent-human encounters are on the rise with continuous demolition of rat habitats [1]. With increased human-rat contact comes an increased risk of exposure to the external parasites and pathogens they harbor. Some external parasites that brown rats carry are harmless, while others are potential vectors for zoonotic pathogens [2]. Common agents include *Yersinia pestis* [3], *Borrelia burgdorferi* [4], and *Bartonella henselae* [5].

There is a reported average of seven cases of the plague annually in the United States with the most common manifestation of *Y. pestis* infection being the bubonic form [6]. Centers for Disease Control and Prevention (CDC) states there is an average of 30,000 cases of Lyme disease diagnosed and reported to the CDC [7]. An estimated 20,000 cases of *B. henselae* are reported in the US annually, according to the CDC [8]. All mentioned pathogens can be transmitted by some of the external parasites that utilize the brown rat as part of their life cycle.

There is a paucity of published research on the prevalence of ectoparasites on *R. norvegicus* in the Caribbean region. This study aimed to estimate the prevalence of external parasites on brown rats from Grenada, West Indies.

## Materials and Methods

### Ethical approval

The project was approved by the Institutional Animal Care and Use Committee (IACUC #16009-R) of St. George’s University (SGU), Grenada.

### Study area

Grenada is a southern Caribbean island with an area of 348.5 km^2^. The country, with low hills, small trees, shrubs, and a tropical climate, is suitable for rats. The country is composed of six parishes: St. Andrew, St. David, St. George, St. John, St. Mark, and St. Patrick. Landscape and climatic conditions are similar in all parishes. The populous parishes of St. David and St. George were selected for this study.

### Collection of rats and ectoparasite samples

One hundred sixty-eight rats were collected live from May to July 2017 using wood and wire mesh traps (45 cm l×15 cm w×15 cm h) baited with cheese or fruit. Traps were placed within 10 m of residential buildings twice weekly in the evening and collected the next morning. Species identification of the captured rats was achieved by evaluating their morphological characteristics. Attributes used to identify the brown rat include coat color, ear stature and color, and length of tail. Brown rats are stout in build with brown-gray coats, prominent bare ears that protrude past the head, and short tails in relation to the length of their body [9]. Traps with rats were covered with black cloth, transported to the Necropsy Laboratory of the SGU School of Veterinary Medicine and transferred to the anesthesia machine. Rats were euthanized using 1-2% isoflurane in oxygen through DRE VP3 anesthesia vaporizer (Avante Health Solutions Company, USA).

The euthanized rats were examined for their physical health, weight, and sex. Rats below 100 g were grouped as a juvenile and over 100 g as an adult following the methodology used by Panti-May *et al*. [10]. Ectoparasites were collected from rats using a fine comb. Fine thumb forceps were also used to remove the parasites from the skin of the rats. Tape was applied to rats to remove parasites that may be present in hair follicles. The collected ectoparasites from individual rats were preserved in 70% ethanol. Paper tape samples were evaluated under a dissection microscope at 400× magnification as described by Parkinson *et al*. [11]. External parasites were identified based on morphological characteristics and identifications described by Richard and David [12] using 30-400× magnification by microscopy.

## Results

Rats are known to normally harbor fleas, mites, and lice [12,13]. In the present investigation, we recorded four taxa of ectoparasites (fleas, mites, lice, and ticks) in brown rats from Grenada. Results of positive rats and ectoparasites are presented in Table-1.

**Table 1:** Distribution of ectoparasites in brown rats from Grenada.

Particulars	(Fur mites) Atopomelidae and Listrophoridae	(Mites) Mesostigmata	(Lice) *Polyplax* ssp.	(Fleas) *Ctenocephalides felis*	(Tick) *Argasidae*	(Mite) *Myobia* spp.
Positive rats with ectoparasite	100/149 (67.1%)	126/149 (84.6%)	10/149 (6.7%)	5/149 (3.4%)	1/149 (0.7%)	1/149 (0.7%)
Total number of ectoparasites	1427	744	13	7	1	1

**Figure-1:**
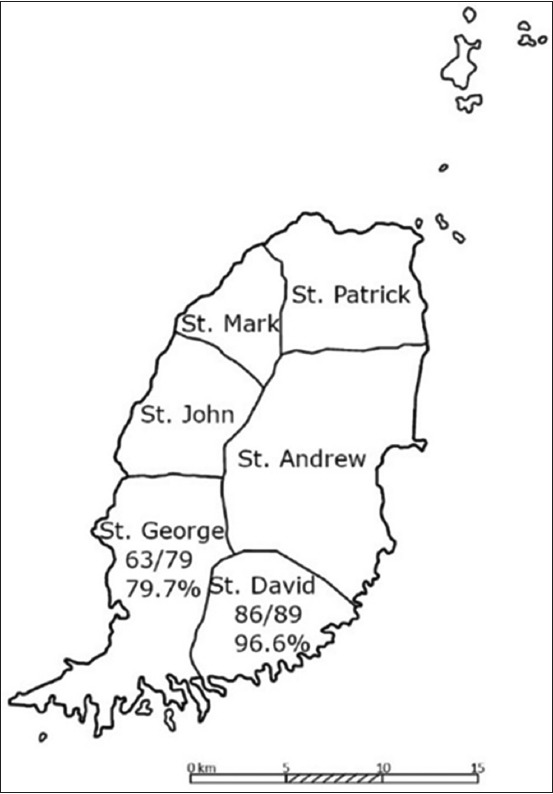
Parishes of Grenada showing study area.

The prevalence of ectoparasites on brown rats was evaluated in two human densely populated parishes in Grenada: St. David and St. George (Figure-1). Out of 168 examined rats, we found 149 (88.7%) infested with ectoparasites. Over 2000 ectoparasites were collected from the 149 infested rats. Parasite prevalence was as follows: mesostigmatid mites (126/149; 84.6%; 5.9 per infested rat), fur mites in the families Atopomelidae and Listrophoridae (100/149; 67.1%; 14.3 per infested rat), *Polyplax* spp. lice (10/149; 6.7%; 1.3 per infested rat), fleas (5/149; 3.4%; 1.4 per infested rat), an unidentified larval tick (1/149; 0.7%; one tick per infested rat), and a mite in the family Myobiidae (1/149; 0.7%; one mite per infested rat).

Rats in St. George Parish had an infestation rate of 79.7% (63/79), while rats in St. David had a 96.6% (86/89) infestation rate (difference: p=0.001). The prevalence in male and female rats in St. George and St. David is included in Table-2. In total, 93.3% (83/89) of male rats and 83.5% (66/79) of female rats were infested with parasites (p=0.054).

**Table 2:** Prevalence of ectoparasites on brown rats from Grenada (parish, sex, and age-wise).

Parish	Total rats (%)	Total rats (%)	Sex (%)		Age (%)	
			
	Positive rats/All rats	Positive rats/All rats	Positive rats/All rats		Positive rats/All rats	
	
			M	F	Adult	Juvenile
St. David	89/168 (53.0)	86/89 (96.6)	48/49 (98.0)	38/40 (95.0)	57/60 (95.0)	7/7 (100)
St. George	79/168 (47.0)	63/79 (79.7)	35/40 (87.5)	28/39 (71.8)	40/47 (85.1)	7/14 (50.0)
Total	168 (100)	149/168 (88.7)	83/89 (93.3)	66/79 (83.5)	97/107 (90.7)^a^	14/21 (66.7)^a^

a For age totals, not all rats were assessed for age. Total rats aged were 128.

## Discussion

Previous researchers reported wide variations in the prevalence of positive brown rats for ectoparasites in different parts of the world. Reports from the following countries demonstrate the diversity in external parasite prevalence from Croatia 32.2% [14]; from Nigeria 9.4% and 38% [15,16]; and from Iran 40.3% [17]. The explanation for differences in ectoparasite prevalence found by previous researchers is not well explained. Soulsby [18] and Paul *et al*. [15] linked the differences to local ecological conditions.

The most numerous ectoparasite present on rats were mesostigmatid mites, which were found on 84.6% of positive rats. Some mesostigmatid mites are of a human health concern as they act as vectors in the transmission of zoonotic pathogens [19]. A study in Egypt collected and tested DNA extracted from 616 tropical rat mites and found one *Bartonella* and two *Rickettsia* spp. in eight pools of tropical fur mites (*Ornithonyssus bacoti*) [20]. It is also a suspected vector for the causative agents of murine typhus (*Rickettsia typhi*) and tularaemia (*Francisella tularensis*) [21].

The second most prevalent ectoparasites found were mites of the family Atopomelidae and Listrophoridae, which were present on 67.1% of infested rats. These fur mites are obligate parasites found attached to the hair shafts of rats. Mites of family Atopomelidae and Listrophoridae feed on sebaceous secretions and tissue particles [22,23]. They are commonly found on hosts in tropical and subtropical climates.

Lice (*Polyplax* spp.) were found on six of the positive examined rats. These ectoparasites are reservoirs and are adept at spreading different zoonotic pathogens. In a Taiwan study, 11 out of 81 collected *Polyplax* spp. collected from *R. norvegicus* were PCR positive for *Bartonella* DNA [24]. Research conducted in Australia on nonindigenous rodents including *R. norvegicus* revealed that 21.4% of rats were positive for external parasites. *Polyplax* spp. made up 23.5% of the external parasites. There are concerns that *Polyplax* spp. are vectors for zoonotic agents, such as *Rickettsia* spp. [25].

During our study, fleas (*Ctenocephalides felis*) were found on 3.4% of rats. Fleas transmit flea-borne spotted fever caused by *Rickettsia felis* [26]. This zoonotic disease has not been established in Grenada, but Kelly *et al*. [27] reported PCR positive rats for *R. felis* in St. Kitts, a neighboring island of Grenada, suggesting that the bacteria are present in cat fleas in the Caribbean. The cat flea is also known to transmit *Bartonella* spp. to humans [28]. The study of the relationship with fleas and their rodent hosts is important as many rodent-associated fleas have been found to harbor more zoonotic species of *Bartonella* [29].

One mite from the family Myobiidae was found on a rat (0.7%). These mites can be found anywhere on the rodent body but are found mainly in dense areas along hair bases [30]. *Myobia* spp. mites feed on skin secretions and interstitial fluids of their host, but not on host blood [19]. These mites are commonly found on mice and rarely on rats. *Myobia* spp. mites may cause mild skin lesions or pruritus, alopecia, and ulceration but have no known association with the transmission of zoonotic diseases [30].

One rat (0.7%) was infested with a soft red larval tick from the family Argasidae. Soft ticks are found in close proximity of humans and animals in arid and semi-arid regions. There are 193 described species, 87 of which are found in the Caribbean, the South of Mexico, and in South America [31]. Interestingly, *R. norvegicus* is not commonly infested with ticks [32]. Many zoonotic tick-borne pathogens are transmitted by ticks in the family Ixodidae, including *Borrelia* spp. (the causative agents of Lyme disease and tick-borne relapsing fever), *Anaplasma* spp. (human granulocytic anaplasmosis, bovine anaplasmosis, and canine infectious cyclic thrombocytopenia), and *Ehrlichia* spp. (ehrlichiosis) [33]. Previous studies described the presence of *Anaplasma* spp. and *Ehrlichia* spp. in Grenada [34,35].

With regard to the infestation rate by sex, our findings corroborate those reported by Paul *et al*. [15] and Stojcevic *et al*.[14] who reported no significant difference with sex. Soliman *et al*. [13] claimed a higher prevalence of ectoparasites in male rats. Contrary to the finding of Soliman *et al*. [13], Webster and Macdonald [36] found significantly more female rats infested with lice and more male rats with mites. In the present study, adult rats had 90.7% prevalence compared to 66.7% juvenile rats (p=0.008). Similar results were reported by Paul *et al*. [15] who found a higher prevalence in adult rats (5.9%) compared to juvenile (3.5%). Soliman *et al*. [13] also observed higher infestations in adults. More research is necessary to explain the relation of sex and age with the presence of ectoparasites on brown rats.

## Conclusion

This study of ectoparasites on brown rats (*R. norvegicus*) in Grenada revealed the presence of four taxa of ectoparasites: mites, lice, fleas, and ticks. Several of these ectoparasites are a potential public health concern as they may serve as vectors of many zoonotic pathogens. With the rise in rodent-human contact due to the urbanization of the brown rat’s habitats, these external parasites should be studied more. Accordingly, upcoming studies by our research group will examine the presence of pathogens in rat ectoparasites in Grenada. The Grenadian population should be educated regarding the health hazards associated with close contact with brown rats. Education of the public will be beneficial in implementing proper rat control in residential areas.

## Author’s Contribution

RNS: planned, supervised the research, and finalized the manuscript. KNT and NFR: collected and identified parasites from brown rats. KNT: drafted the manuscript. DMF: helped KNT and NFR in identification of ectoparasites and finalizing the manuscript. CCS and RDP: confirmed identity of ectoparasites. KT: collection of rats and helped KNT and NFR in collection of parasites. All authors read and approved the final manuscript.
